# The Effects of Statins on Respiratory Symptoms and Pulmonary Fibrosis in COVID-19 Patients with Diabetes Mellitus: A Longitudinal Multicenter Study

**DOI:** 10.1007/s00005-023-00672-1

**Published:** 2023-02-28

**Authors:** Mohammadamin Sadeghdoust, Farnaz Aligolighasemabadi, Tania Dehesh, Nima Taefehshokr, Adel Sadeghdoust, Katarzyna Kotfis, Amirhossein Hashemiattar, Amir Ravandi, Neda Aligolighasemabadi, Omid Vakili, Beniamin Grabarek, Rafał Staszkiewicz, Marek J. Łos, Pooneh Mokarram, Saeid Ghavami

**Affiliations:** 1 Department of Internal Medicine, Mashhad Medical Sciences Branch, Islamic Azad University, Mashhad, Iran; 2 Department of Biostatistics and Epidemiology, School of Public Health, Kerman University of Medical Sciences, Kerman, Iran; 3 Department of Microbiology and Immunology, Center for Human Immunology, The University of Western Ontario, London, ON Canada; 4 Department of Internal Medicine, School of Medicine, Hormozgan University of Medical Sciences, Bandar Abbas, Iran; 5 Department of Anesthesiology, Intensive Therapy and Acute Intoxications, Pomeranian Medical University in Szczecin, Szczecin, Poland; 6 Department of Radiology, Mashhad Medical Sciences Branch, Islamic Azad University, Mashhad, Iran; 7 Institute of Cardiovascular Sciences, Sr. Boniface Research Centre, University of Manitoba, Winnipeg, Canada; 8 Department of Internal Medicine, School of Medicine, Razi Hospital, Guilan University of Medical Sciences, Rasht, Iran; 9 Department of Clinical Biochemistry, School of Pharmacy and Pharmaceutical Sciences, Isfahan University of Medical Sciences, Isfahan, Iran; 10 Department of Histology, Cytophysiology and Embryology, Faculty of Medicine in Zabrze, Academy of Silesia in Katowice, Zabrze, Poland; 11 Department of Gynaecology and Obstetrics, Faculty of Medicine in Zabrze, Academy of Silesia in Katowice, Zabrze, Poland; 12 Laboratory of Molecular Biology and Virology, GynCentrum, Katowice, Poland; 13 Department of Neurosurgery, 5Th Military Clinical Hospital with the SP ZOZ Polyclinic in Krakow, Krakow, Poland; 14 Biotechnology Center, Silesian University of Technology, Gliwice, Poland; 15 Autophagy Research Center, Department of Biochemistry, Shiraz University of Medical Sciences, Shiraz, Iran; 16 Department of Human Anatomy and Cell Science, Rady Faculty of Health Sciences, Max Rady College of Medicine, University of Manitoba, Winnipeg, Canada; 17 Faculty of Medicine in Zabrze, University of Technology in Katowice, Academia of Silesia, Zabrze, Poland; 18 Research Institute of Oncology and Hematology, Cancer Care, Manitoba University of Manitoba, Winnipeg, Canada; 19 Biology of Breathing Theme, Children Hospital Research Institute of Manitoba, University of Manitoba, Winnipeg, Canada

**Keywords:** COVID-19, Diabetes mellitus, Statins, Post-acute COVID-19 syndrome, Pulmonary fibrosis

## Abstract

The aim of this prospective cohort study was to explore the effect of statins on long-term respiratory symptoms and pulmonary fibrosis in coronavirus disease 2019 (COVID-19) patients with diabetes mellitus (DM). Patients were recruited from three tertiary hospitals, categorized into Statin or Non-statin groups, and assessed on days 0, 28, and 90 after symptoms onset to record the duration of symptoms. Pulmonary fibrosis was scored at baseline and follow-up time points by high-resolution computed tomography scans. Each group comprised 176 patients after propensity score matching*.* Data analysis revealed that the odds of having cough and dyspnea were significantly higher in the Non-statin group compared to the Statin group during the follow-up period. Overall, there was no significant difference in the change in pulmonary fibrosis score between groups. However, Non-statin patients with > 5 years of DM were more likely to exhibit a significantly higher fibrosis score during the follow-up period as compared to their peers in the Statin group. Our results suggest that the use of statins is associated with a lower risk of developing chronic cough and dyspnea in diabetic patients with COVID-19, and may reduce pulmonary fibrosis associated with COVID-19 in patients with long-term (> 5 years) DM.

**Graphical Abstract:**

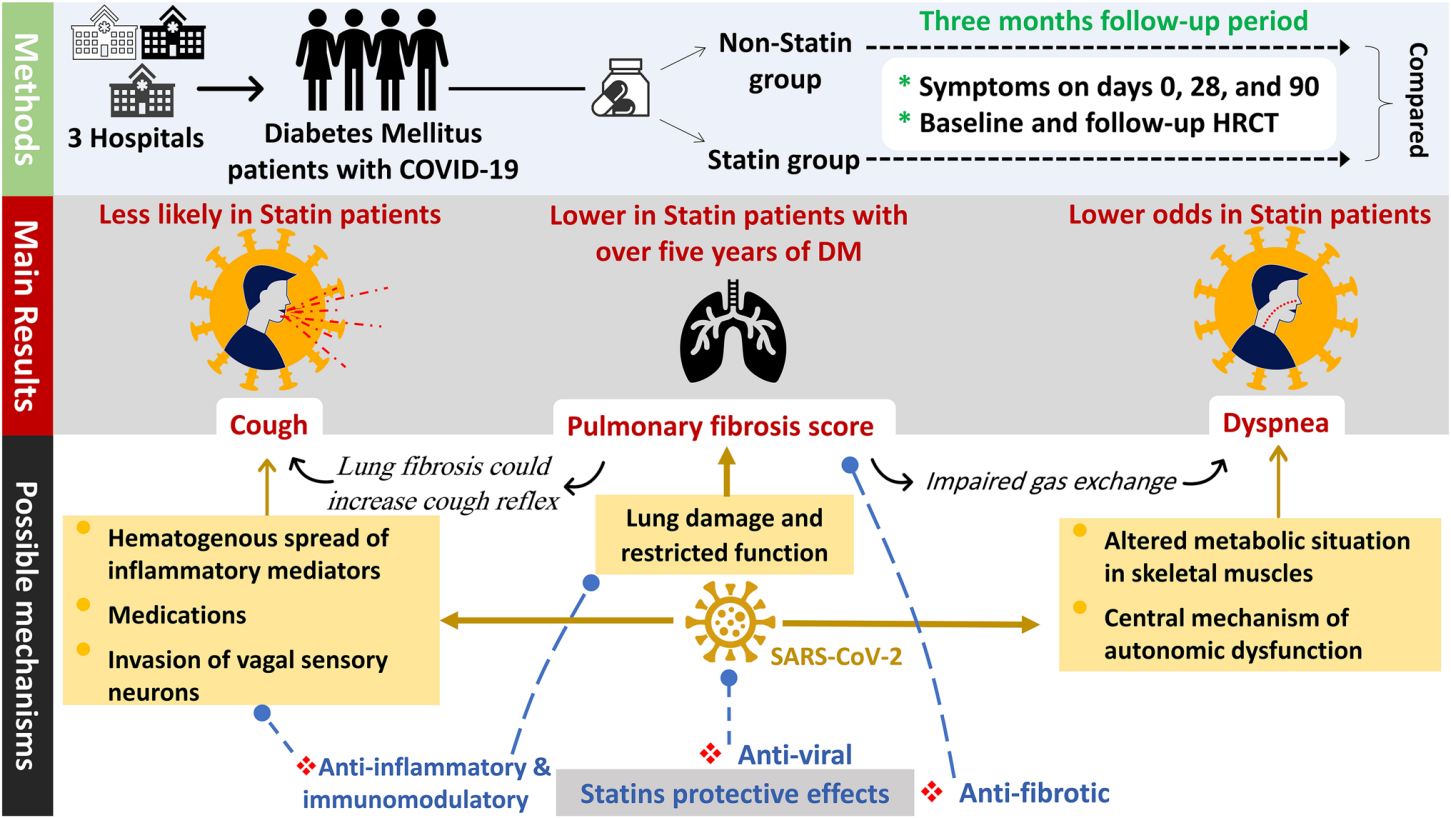

## Introduction

Coronavirus disease 2019 (COVID-19) caused by severe acute respiratory syndrome coronavirus 2 (SARS-CoV-2) has spread uncontrollably around the world with considerable impacts on public health and the international economy (Peterson and Walker 2022; Walls et al. 2020). This high virulence is due to multiple mechanisms allowing SARS-CoV-2 to manipulate host immune responses, thus prolonging viral clearance periods in patients (Taefehshokr et al. 2020). The virus primarily targets the respiratory system and mainly enters respiratory cells by binding to cell surface receptor proteins such as angiotensin-converting enzyme 2 (ACE2) and neuropilin-1 (Kouhpayeh et al. 2021; Kyrou et al. 2021; Shojaei et al. 2020b; Siri et al. 2021; Walls et al. 2020). ACE2 is recognized as a non-immune receptor for SARS-CoV-2 and binds to the viral S protein receptor-binding motif at its N-terminal extracellular catalytic domain (Gawish et al. 2022; Jackson et al. 2022). Most COVID-19 patients present with mild to moderate symptoms but roughly one-sixth develop severe pneumonia, of which approximately 5% ultimately develop acute respiratory distress syndrome, septic shock, or multiple organ failure (Centers for Disease Control and Prevention 2020; Peymani et al. 2021). Evidence suggests that some patients experience long-term symptoms and pulmonary fibrosis after recovery from the acute phase of COVID-19 has emerged; an undesirable pathologic phenomenon known as long COVID or post-acute COVID-19 syndrome (PCS) (Jutant et al. 2022). PCS is affected by multiple factors such as dysregulated inflammation, organ damage, and the presence of certain pre-existing conditions, including diabetes mellitus (DM) (Habibzadeh et al. 2021; Raveendran and Misra 2021).

DM is a common underlying disease in COVID-19 patients and one of the leading causes of morbidity and mortality worldwide (Centers for Disease Control and Prevention 2020, 2022; Drozdzal et al. 2021; Guo et al. 2020). COVID-19 might put DM patients at risk of hyperglycemia, which consequently might modulate immune and inflammatory responses (Lim et al. 2021). Furthermore, the dysregulated immune system and pro-inflammatory state in DM, characterized by excessive and improper cytokine responses, might predispose COVID-19 patients to severe respiratory symptoms, organ damage, and poor clinical outcomes (Hussain et al. 2020; Lim et al. 2021). Thus, DM could augment the prolonged inflammatory response in COVID-19, thereby potentially promoting pulmonary fibrosis that can lead to long-term respiratory symptoms as seen in PCS patients (Raveendran and Misra 2021). In addition, there are several common main risk factors for severe COVID-19 and idiopathic pulmonary fibrosis that include increasing age, male sex, and associated comorbidities such as DM (George et al. 2020; Lechowicz et al. 2020). Therefore, the control of DM by glucose-lowering medications and the prevention of disease complications by lipid-lowering agents such as statins need special attention in COVID-19 patients.

Statins are well-known cholesterol-lowering medications recommended by the American Diabetes Association for nearly all DM patients (Ahmadi et al. 2020; American Diabetes Association Professional Practice Committee 2022). Their principal mechanism of action is blocking the mevalonate pathway by competitively inhibiting 3-hydroxy-3-methylglutaryl coenzyme A reductase (Adhyaru and Jacobson 2018; Shojaei et al. 2020a). Beyond their lipid-lowering effects, these agents show pleiotropic immunomodulatory, anti-inflammatory, anti-fibrotic, and anti-cancer effects (Ahmadi et al. 2020; Alizadeh et al. 2017; Emami et al. 2019; Liao and Laufs 2005; Schaafsma et al. 2011a). Moreover, statins suppress HIV replication through upregulation of p21 in CD4 T cells (Elahi et al. 2016), and inhibit respiratory syncytial virus replication as well as load in mice (Gower and Graham 2001), indicating anti-viral properties. Statins could potentially limit the exaggerated inflammatory response by amplifying ACE2 expression and inhibiting Toll-like receptor nuclear factor κB and NOD-like receptors family pyrin domain containing 3 inflammasomes (Drozdzal et al. 2021; Lee et al. 2020). Recent investigations have shown strong evidence for the anti-fibrotic effects of statins in airway resident cells and improved clinical outcomes in idiopathic pulmonary fibrosis patients using statins (Kreuter et al. 2017; Schaafsma et al. 2011b; Watts et al. 2005). Moreover, statins may potentially affect COVID-19 pathogenesis via targeting autophagy and apoptosis of host cells and virulence of SARS-CoV-2 (Han et al. 2018; Peng et al. 2018). Hence, the use of statins has attracted much attention as an adjunctive therapy to mitigate dysregulated inflammation and improve the clinical outcomes of COVID-19 patients (Scheen 2021).

Based on the scale of the pandemic, the health burden of PCS and fibrotic lung disease following COVID-19 is likely to be high. At the same time, despite the scientific rationale for using statins in COVID-19 patients, the effects of statins on long-term respiratory symptoms and pulmonary fibrosis have not been characterized yet. Therefore, we followed up on our previous retrospective investigation on the impact of statins on COVID-19 (Peymani et al. 2021) and designed this prospective cohort study to specifically evaluate the effects of statins on the duration of respiratory symptoms and changes in pulmonary fibrosis using high-resolution computed tomography in COVID-19 patients with DM over a three-month follow-up period.

## Materials and Methods

### Study Design and Patients

This multi-center prospective cohort study was conducted between May and December 2021 in three tertiary hospitals in Iran: The Karoon Hospital (Gotvand city), Razi Hospital (Rasht city), and Golestan Hospital (Ahvaz city). This study was conducted at the same time as the fourth and fifth waves of the COVID-19 pandemic, and based on the available data the delta variant was becoming the dominant strain in that period of time (Yavarian et al. 2022). This work was approved by the Shiraz University of Medical Sciences (IR.SUMS.REC.1399.151) and the Institutional Review Board of the relevant centers. Written informed consent was obtained from all the participants.

COVID-19 patients with diabetes who met the inclusion criteria were included in Statin or Non-statin groups and followed up for three months after initial symptoms to assess the potential effects of statins on long-term respiratory symptoms and pulmonary fibrosis. Inclusion criteria were: (a) 18 < age (years) < 85; (b) confirmed diagnosis of diabetes mellitus based on American Diabetes Association guidelines (American Diabetes Association 2021); (c) confirmed diagnosis of COVID-19 defined as a laboratory-confirmed SARS-CoV-2 infection through real-time reverse-transcriptase polymerase chain reaction; (d) presenting with at least one of the following respiratory symptoms: cough, dyspnea, chest discomfort, anosmia, ageusia, fever, sweating, fatigue, myalgia, arthralgia, or headache. Patients with chronic respiratory disease, active hepatic disease, deafness, blindness, intellectual disability, and critical cases were excluded.

### Baseline Assessment and Follow-Up

Baseline demographics, comorbidities, and blood laboratory test results were collected from the electronic medical records systems during the first visit. Initial signs, symptoms, and the presence of abnormal sounds in auscultation were also recorded. Modified Medical Research Council (mMRC) Dyspnea Scale and cough symptom score (CSS) were used to score the severity of dyspnea and cough, respectively.

All patients were offered two follow-up interviews on days 28 and 90 after presenting initial symptoms on day 0. Additionally, a clinic follow-up card was given to each patient to record the exact initiation and end date of symptoms. Also, patients with available baseline and follow-up high-resolution computed tomography scans (HRCTs) were included in HRCT analysis to evaluate pulmonary fibrosis.

### Review of HRCT Images

Pulmonary fibrosis in HRCT images was scored from 0 to 30 based on a method described by Camiciottoli et al. (2007). Briefly, the total score is equal to the score for all types of lesions (ground-glass opacities = 1; linear opacities = 2; interlobular septal thickening = 3; reticulation = 4; honeycombing and bronchiectasis = 5) plus the extent score for each type of lesions (1–3 involved pulmonary segments = 1; 4–9 segments = 2; more than 9 segments = 3). All images were reviewed randomly by an expert radiologist and an experienced research assistant, who were blinded to the study groups.

### Statistical Analysis

Propensity score matching was performed through a 1:1 greedy matching algorithm to limit potential residual confounding factors. In observational studies, it is impossible to have control over confounder variables at the beginning of the study. Therefore, confounder effects should be removed by matching. Covariates in the propensity analyses included age, sex, obesity, Charlson comorbidity index, smoking status, use of insulin, diabetes duration, serum level of glycosylated hemoglobin, history of liver disease, renal disease, hypertension, cardiovascular disease, and cerebrovascular disease.

Continuous data are reported as mean and standard deviation (SD) or median and interquartile range [IQR], and categorical data are shown as numbers and percentages. The χ^2^ test, Student *t *test, and Mann–Whitney *U* tests were used for comparative analysis of baseline characteristics. Using the Kaplan–Meier (log-rank) test, patients in two groups were compared in terms of time to becoming symptom-free. On bivariate analysis, odds ratios along with their 95% confidence intervals (95%CIs) were calculated using a marginal model via generalized estimation equation. Marginal models are substitutions of repeated measurement analysis in follow-up studies when the response variable does not have a normal distribution. We also conducted a subgroup analysis to explore how statins in combination with certain factors affect pulmonary fibrosis.

IBM SPSS Statistics (IBM Corporation, version 19.0) and GraphPad Prism software version 8.0.2 (GraphPad Software, San Diego, California, USA), R version (4.1.0) were used to perform data analysis. Differences were considered statistically significant when *p*-values ≤ 0.05.

## Results

### Participants

**Fig. 1:**
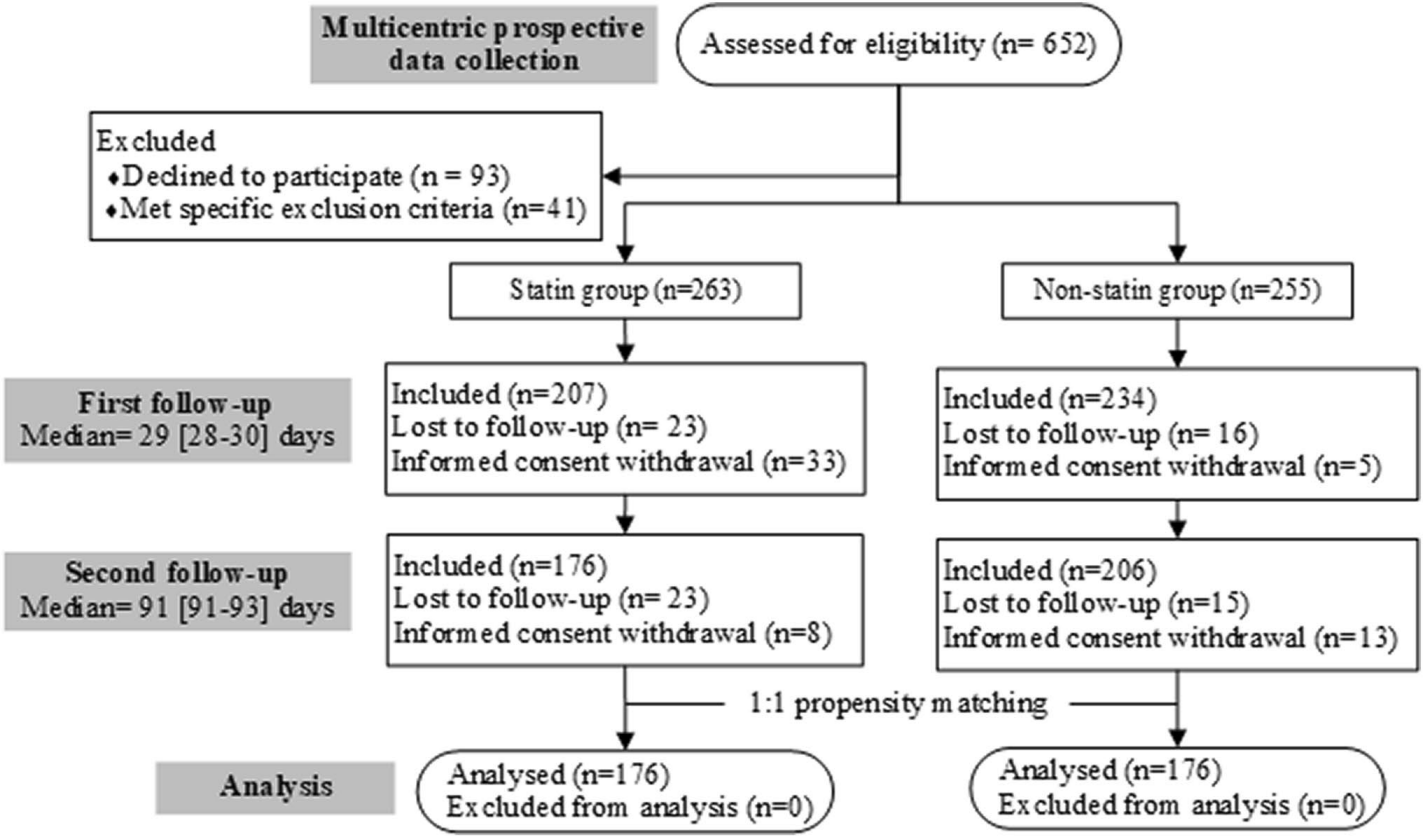
Flow diagram of enrollment and follow-up of diabetic patients with COVID-19 in the statin and non-statin groups

A total of 652 diabetic patients with confirmed COVID-19 was assessed for participation eligibility (Fig. 1). After excluding 134 patients, 518 patients were included in the study. A hundred and seventy-six out of 263 patients in the Statin group and 206 out of 255 patients in the Non-statin group successfully attended the first and second follow-up interviews. After propensity matching, 176 patients from each group were included in the data analysis.

Table 1 summarizes the demographics, clinical backgrounds, and laboratory test results of patients in each group. Females with controlled diabetes were the dominant population, while hypertension was the most common comorbidity. Patients in the Statin group had significantly lower serum levels of LDL cholesterol, triglycerides, and platelets.

**Table 1:** Demographics and baseline characteristics of diabetic patients infected with SARS-CoV-2

Characteristic	Non-statin (*n* = 176)	Statin (*n* = 176)	*p*-Value
Female	120 (68.2)	128 (72.7)	0.41
Age, years	61 [54–66]	62 [56–66]	0.21
Body mass index, kg/m^2^	28.3 (3)	28.7(2.8)	0.16
Smoker	23 (13.1)	20 (11.4)	0.62
Duration of diabetes, years	4 [3–7]	5 [4–7]	0.12
Poor-controlled diabetes*	58 (33)	43 (24.4)	0.077
Number of comorbidities	2 [1–2]	2 [1–2]	0.15
Hypertension	87(49.4)	99 (56)	0.24
Cardiovascular disease	25 (14.2)	36 (20.5)	0.005
Cerebrovascular disease	9 (5.1)	14 (8)	0.38
Chronic kidney disease	24 (13.6)	26 (14.8)	0.43
Liver disease	10 (5.7)	12 (6.8)	0.66
Charleston comorbidity index score	4.4 (1.2)	4.6 (1.5)	0.22
Laboratory tests			
White blood cell count; × 10^9^/L	8.1 [6.6–9.3]	7.3[6.4–8.6]	0.07
Neutrophil count; × 10^9^/L	6.1 [5.1–7.2]	5.7 [4.9–6.6]	0.034
Lymphocytes count; × 10^9^/L	1.4 [1.1–1.8]	1.4 [1.1–1.8]	0.81
Platelets count; × 10^9^/L	284 [235–350]	270 [214–321]	0.028
Haemoglobin; g/L	12.1 [11.3–13]	11.8 [10.8–12.7]	0.061
Serum creatinine; µmol/L	1.1 [0.9–1.2]	1.09 [1–1.27]	0.22
Triglyceride; mg/dL	157 [134–182]	153 [126–182]	0.01
Cholesterol; mg/dL	172 [155–197]	168 [150–189]	0.12
LDL; mg/dL	95 [76–116]	89 [60–104]	0.001
HDL; mg/dL	32 [25–40]	35 [23–42]	0.19
Aspartate aminotransferase; U/L	25.6 [17.8–39.3]	29.4 [19.3–44.6]	0.17
Alanine aminotransferase; U/L	19.3 [15.2–30.7]	22.8 [18.1–31.9]	0.19
ESR	37 [26.2–51.7]	32 [22–46.5]	0.089
d-dimer, µg/mL	0.5 [0.4–0.7]	0.5 [0.4–1.1]	0.18
C-reactive protein; mg/dL	12.7 [11.7–35]	23 [11.2–37]	0.99
HbA1c	7.9 [6.7–7.4]	8.1 [7.5–9.5]	0.082
Medications			
Atorvastatin	NA	148 (84.1)	NA
Rosuvastatin	NA	17 (9.7)	NA
Simvastatin	NA	8 (4.5)	NA
Other statins	NA	3 (1.7)	NA
Insulin	64 (36.4)	60 (34.1)	0.73
Metformin	98 (55.7)	86 (48.9)	0.24
Other oral diabetes medications	53 (30.1)	60 (34.1)	0.42
Antihypertensive of any type	73 (41.5)	80 (45.5)	0.51
Anti-coagulant and anti-platelet	62 (35.2)	57 (32.4)	0.65
NSAIDs	69 (39.2)	74 (42)	0.66
Proton pump inhibitors/antacids	51 (29)	59 (33.5)	0.42

Data are presented as mean ± standard deviation, median [inter quartile range], or number (percentage)

*NSAIDs* non-steroidal anti-inflammatory drugs; *NA* not applicable

*HbA1c value is 7% or higher

### Respiratory Symptoms

The frequency of occurring respiratory symptoms is summarized in Table 2. Cough was the most common initial symptom in both groups, followed by fever/sweating and dyspnea. Cough in the Statin group dropped from 60.2% to 11.4% and 5.7% on days 28 and 90 of follow-up, respectively. In patients who did not receive statins, cough prevalence decreased from 51.7% to 17% and 5.7%. Similarly, there was a dramatic reduction in the presence of dyspnea and other symptoms throughout the follow-up period. Further data analysis revealed that the odds of having a cough during the follow-up period were higher in patients not using statins compared to those who did (OR: 1.35, CI 95%: 1.01–1.81; *p* = 0.046). In addition, patients in the Non-satin group were more likely to present with dyspnea (OR:1.42, CI 95%: 1.01–1.81; *p* = 0.046). However, there were no statistically significant differences in experiencing other symptoms between groups throughout the follow-up period.

**Table 2:** The frequency of initial and persistent symptoms, and the results of marginal model (GEE estimation) analysis in statin and non-statin patient groups

	Onset (Day 0)		Day 28		Day 90		GEE estimation (statin vs non-statin)		
Symptoms	N	S	N	S	N	S	OR	95% CI for OR	*p*-value
Fever/sweating	105 (59.7)	91 (51.7)	43 (24.4)	33 (18.8)	21 (11.9)	16 (9.1)	1.29	0.94–1.78	0.11
Fatigue	89 (50.6)	79 (44.9)	39 (22.2)	33 (18.8)	12 (6.8)	7 (4)	1.24	0.91–1.71	0.18
Myalgia/arthralgia	50 (28.4)	56 (31.8)	24 (13.6)	26 (14.8)	7 (4)	9 (5.1)	0.86	0.57–1.31	0.49
Headache	48 (27.3)	61 (34.7)	21 (11.9)	25 (14.2)	10 (5.7)	6 (3.4)	0.84	0.55–1.27	0.41
Cough	106 (60.2)	91 (51.7)	30 (17)	20 (11.4)	10 (5.7)	5 (2.8)	1.35	1.01–1.81	0.046
Dyspnea	97 (55.1)	81 (46)	45 (25.6)	36 (20.5)	21 (11.9)	10 (5.7)	1.42	1.02–1.98	0.037
Chest discomfort	48 (27.3)	37 (21)	30 (17)	14 (8)	7 (4)	3 (1.7)	1.58	0.99–2.51	0.052
Anosmia/ageusia	77 (43.8)	87 (49.4)	35 (19.9)	39 (22.2)	19 (10.8)	17 (9.7)	1.24	0.91–1.71	0.18
Abnormal sound on auscultation	76 (43.2)	70 (39.8)	40 (22.7)	25 (14.2)	24 (13.6)	13 (7.4)	0.86	0.57–1.31	0.49

Data are presented as absolute numbers and (percentages); each group contained a total of 176 patients

*CI* confidence interval; *GEE* generalized estimation equation; *N* non-statin group; *OR* odds ration; *S* statin group

**Fig. 2:**
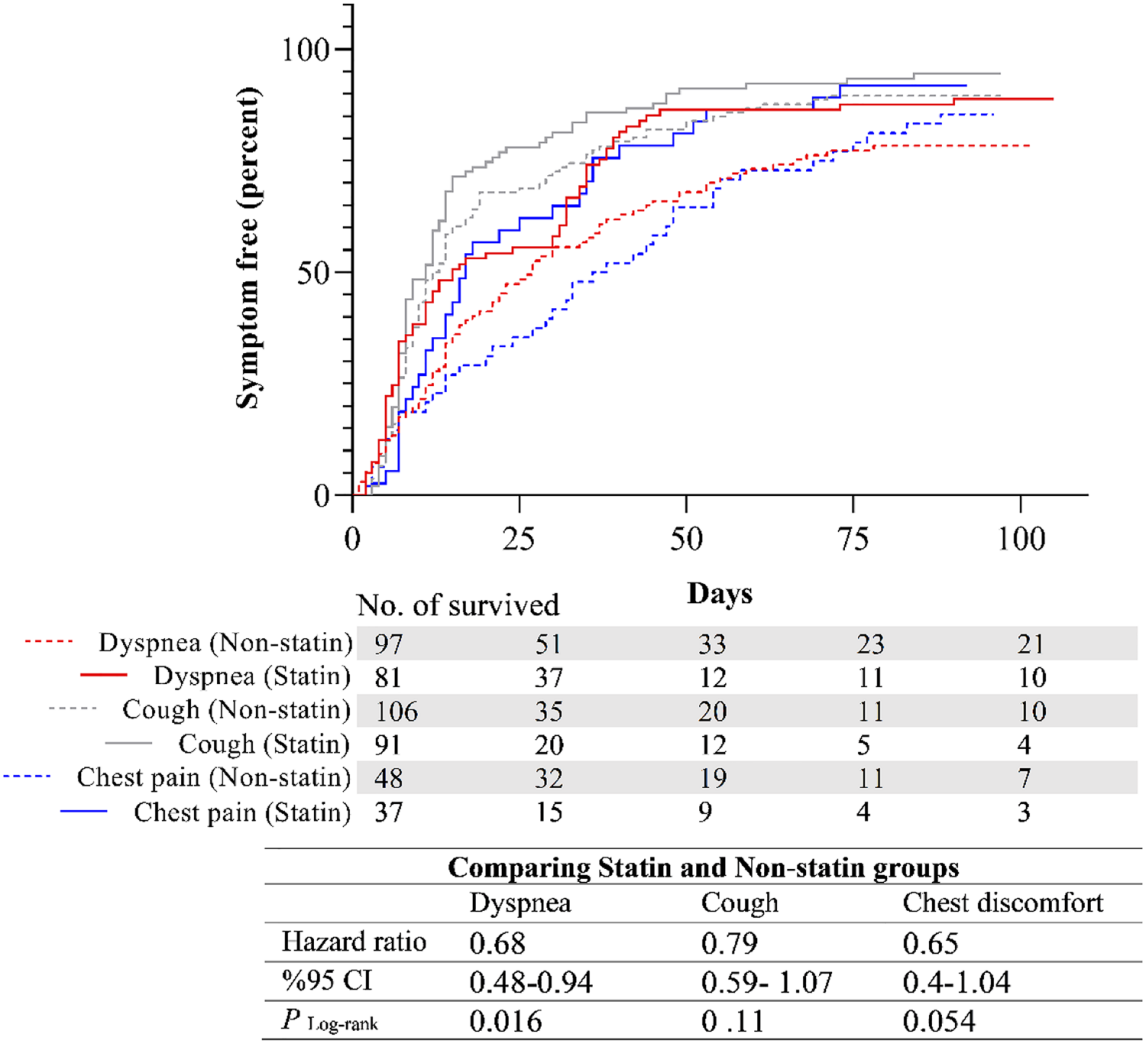
Kaplan–Meier curves showing the symptom-free percentage of patients in each group during the study

Figure 2 shows a Kaplan–Meier analysis of the time to a respiratory symptom-free day in each group (cough, dyspnea, chest pain). The results revealed a trend toward an earlier resolution of cough in the Statin group (HR: 0.68, 95% CI: 0.48–0.94, *p*Log-rank: 0.016). On the other hand, there were no significant differences in time to the first symptom-free day of dyspnea or chest pain symptoms between our study groups.

**Fig. 3:**
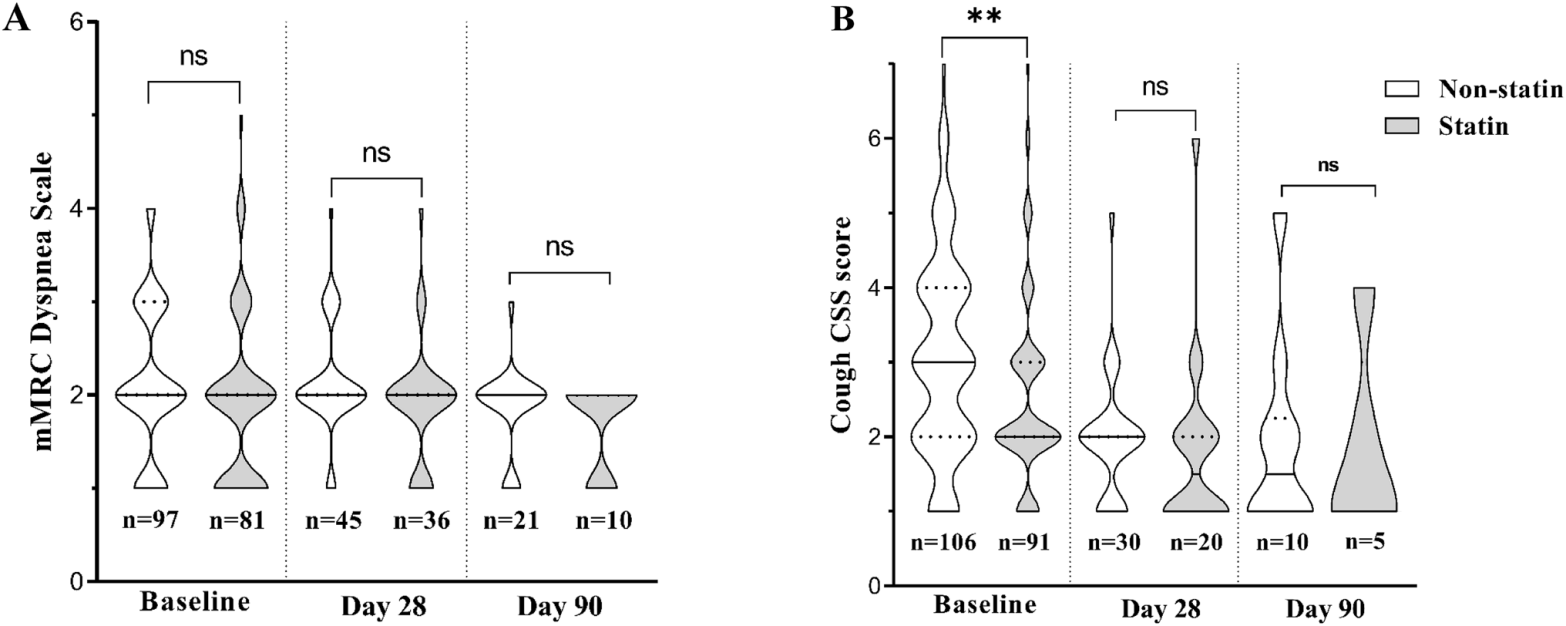
Comparison of the dyspnea (**A**) and cough (**B**) severity in the statin and non-statin groups. Violin plots are showing the distribution of the Medical Research Council (mMRC) dyspnea scale and cough symptom score (CSS) at different time points. Solid and dotted lines represent the median values and quartiles, respectively. **Significant difference (*p* < 0.001); *ns* not significant

The baseline and follow-up severities of cough and dyspnea are shown in Fig. 3A, B. The baseline median (M) cough CSS score of Non-statin patients was 3 (*Q*_1_ = 2, *Q*_3_ = 4), which was significantly higher than in the statin group (*M* = 2, *Q*_1_ = 2, *Q*_3_ = 3; *p* = 0.003). No other significant differences in CSS or mMRC dyspnea severity scores could be observed between groups throughout the follow-up period (*p* > 0.05 for all).

### Pulmonary Fibrosis Scores

**Fig. 4:**
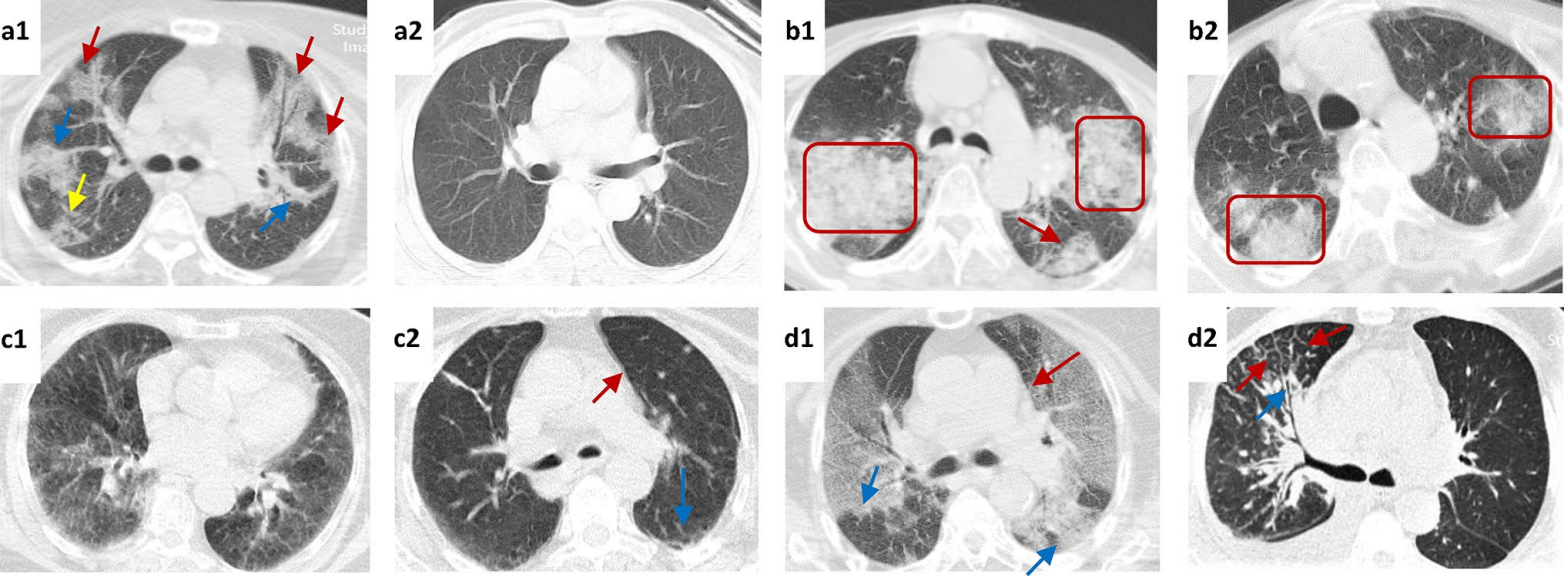
Representative images of axial sections of initial and follow-up HRCT scans of the lungs in COVID-19 patients with diabetes mellitus in statin (**a** and **b**) and non-statin (**c** and **d**) groups. **a1** Initial HRCT image (fourth day of manifestations) of the lungs of a 52-year-old male (Statin group) complaining of dyspnea and cough. The image shows consolidation and ground glass opacity (GGO) in the anterior (red arrows) and posterior segments (blue arrows) of the upper lobes of both lungs, and the superior segment of the lower lobe of the right lung (yellow arrow). **a2** The same patient came in on day 61 with a persistent cough. The follow-up HRCT showed significant regression of consolidation and GGO, without evidence of fibrotic changes. **b1** Initial HRCT image (third day of manifestations) of the lungs of a 64-year-old female (Statin group) presenting with dyspnea shows an extensive airspace consolidation mainly in the posterior segments of the upper lobes in both lungs (red rectangle), diffuse GGOs, and reticular opacities as shown in the superior segment of the lower lobe of the right lung (red arrow). **b2** Although follow-up HRCT images on day 43 show prominent regression of consolidation to GGOs, both lungs still have opacities. The dyspnea improved 32 days after initiation. **c1** Initial HRCT image (third day of manifestations) of the lungs of a 62-year-old female (Non-statin group) presenting with cough and dyspnea revealed diffuse GGOs in both lungs associated with fine linear opacity. **c2** Follow-up HRCT image on the 38th day of manifestations shows linear opacity (red arrow) and subpleural opacity in the superior segments of the bilateral lower lobes (blue arrows). The cough disappeared after two months, but the dyspnea persisted throughout the follow-up period. **d1** Initial HRCT image (fifth day of manifestations) of the lungs of a 59-year-old male (Non-statin group) presenting with fever, cough, and dyspnea. Extensive bilateral GGOs (red arrows) and consolidation are seen. **d2** The second HRCT obtained 82 days later shows interlobular septal thickening (red arrows) and bronchial wall thickening (blue arrow) in the anterior segment of the upper lobe of the right lung and a significant reduction of GGO in both lungs. Dyspnea and cough persisted for 59 and 55 days, respectively

Fifty-one patients in the statin group and 42 in the non-statin group underwent both initial and follow-up HRCTs, which were taken 6 ± 3.2 and 51.9 ± 17.7 days after the onset of symptoms, respectively; HRCT imaging data are listed in Table 3. The most common findings were ground glass opacity, linear opacity, and reticulation in both groups. Most of the cases showed improvement in HRCT features and reduction in the involved segments over the study time course (Fig. 4). The initial median pulmonary fibrosis score was 8 for the non-statin [IQR = 6–12] as well as the statin [IQR = 6–11] group, which dropped to 5 [IQR = 0–8 and IQR = 0–6, respectively] in both groups as assessed in the follow-up HRCTs; no significant differences in pulmonary fibrosis score were observed between groups (β = 1.225, 95% CI = –0.47–2.92; *p* = 0.15).

**Table 3:** Baseline and follow-up HRCT findings in statin and non-statin groups

HRCT features	Non-statin group (*n* = 42)		Statin group (*n* = 51)	
	Initial	Follow-up	Initial	Follow-up
Ground glass opacity	35 (83.3)	20 (47.6)	41 (80.4)	23 (45.1)
Affected segments	6 [3–8]	4 [2.25–6]	6 [4.5–10]	4 [3–6]
Linear opacity	33 (78.6)	20 (47.6)	40 (78.4)	19 (37.3)
Affected segments	4 [3–5.5]	2 [1.25–3]	3.5 [2–5.75]	3 [1–4]
Interlobular septal thickening	15 (35.7)	7 (16.7)	12 (23.5)	4 (7.8)
Affected segments	2 [1–2]	1.5 [1–2]	2 [1–2]	1 [1–2]
Reticulation	17 (40.5)	11 (26.2)	14 (27.5)	7 (13.7)
Affected segments	1 [1–2]	1 [1–1]	1 [1–2]	1 [1–1]
Honeycombing / Bronchiectasis	6 (14.3)	3 (7.1)	6 (11.8)	4 (7.8)
Affected segments	2 [1–2]	1 [1–1]	2 [1–3.25]	1 [1–1]
Pulmonary fibrosis score	8 [6–12]	5 [0–8]	8 [6–11]	5 [0–6]

Data are presented as mean ± standard deviation or median [inter quartile range]

Further subgroup analysis was performed by categorizing participants into subsets based on shared characteristics such as the use of metformin, insulin, non-steroidal anti-inflammatory drugs, duration of DM, and the control status of DM (Table 4). These analyses revealed that Non-statin patients suffering from DM > 5 years were more likely to have a higher fibrosis score during the follow-up period (2.43 scores higher on average, SEM = 3.36) compared to Statin patients with a similar DM history (95% CI = –0.47–2.92, *p* = 0.041).

**Table 4:** Subgroup analyses of HRCTs to explore the effects of statins on pulmonary fibrosis score

Variables		*N*		β	95% CI		*p*-value
		Non-statin	Statin		Upper	Lower	
Metformin	Yes	24	22	1.73	–0.53	3.99	0.134
	No	18	29	0.33	–2.23	2.88	0.801
Insulin	Yes	28	19	1.59	–1.58	4.76	0.325
	No	24	32	0.948	–0.89	2.79	0.314
NSAIDs^*^	Yes	19	22	0.309	–1.84	2.46	0.779
	No	23	29	1.99	–0.47	4.46	0.113
NSAIDs and Metformin	Yes	12	9	1.57	–1.47	4.61	0.311
	No	30	42	1.221	–0.819	3.25	0.238
NSAIDs and Insulin	Yes	5	9	–1/12	–5.26	3.02	0.595
	No	37	42	1/512	–0.316	3.341	0.105
Diabetes duration	> 5 years	13	14	2.43	0.12	4.74	**0.041**
	≤ 5 years	29	37	0.353	–2.62	3.33	0.816
Controlled diabetes	Yes	13	13	0.462	–2.30	3.22	0.743
	No	29	38	1.53	0.57	3.64	0.152

^*^Controlled diabetes is defined as HbA1c value of 7% or higher

*NSAIDs* non-steroidal anti-inflammatory drugs

## Discussion

This multicenter prospective study revealed that the use of statins is associated with lower odds of cough and dyspnea over a three-month follow-up period after the onset of COVID-19 in patients with diabetes. Moreover, patients on statins experienced substantially lower cough severity compared to non-users. Despite the improvement in severity and duration of symptoms, Statin and Non-statin patients showed no significant differences in the improvement of pulmonary fibrosis score as assessed by HRCT, with the exception of statin users suffering from DM > 5 years who exhibited significant improvement in pulmonary fibrosis as compared to non-statin patients with chronic DM. There is a paucity of prospective studies that have assessed the effects of statins on manifestations of COVID-19 or pulmonary fibrosis in DM patients, whereas retrospective studies mainly focused on assessing the mortality rate and reported controversial results. In a French nationwide observational study involving 2449 DM patients hospitalized for COVID-19, routine statin treatment was shown to be significantly associated with increased mortality (Cariou et al. 2021). In contrast, others reported that in-patient statin use was associated with a considerable reduction in the mortality rate of COVID-19 patients with DM (Lohia et al. 2021; Saeed et al. 2020). So, there appears to be a lack of consensus on the impact of statins on clinical outcomes in DM patients with COVID-19. Our current findings provide further evidence for the beneficial effects of statin use in these patients.

We observed positive effects of statins on the frequency, severity, and duration of cough. Cough is distressing to patients, causes social isolation, and increases the risk of community transmission by respiratory droplets (Dhand and Li 2020; Hulme et al. 2019). Chronic cough in PCS might result from different mechanisms such as the hematogenous spread of inflammatory mediators, the use of specific types of medications, the invasion of vagal sensory neurons by SARS-CoV-2, or a neuroinflammatory response leading to peripheral and central hypersensitivity of cough pathways (Song et al. 2021). Furthermore, pulmonary fibrosis could increase cough reflex sensitivity due to mechanical stimulation of the chest wall (Jones et al. 2011). In contrast to a cough that can persist after the flu or a common cold, chronic cough in PCS is often accompanied by other associated presentations, which could indicate a common pathological mechanism such as pulmonary fibrosis (Song et al. 2021). As evident from our results, patients in the non-statins group were more likely to have dyspnea throughout the follow-up period compared to the statin group. Hypothetically, more pronounced or sustained pulmonary fibrosis in non-statin patients (as compared to statin users) could be a possible explanation for the higher frequency of chronic cough and dyspnea in this group.

However, the analysis of follow-up CT images indicated no statistically significant differences in pulmonary fibrosis score between the overall population of the statin and non-statin groups; of note, this could be related to the relatively low number of assessed HRCTs. Interestingly, further subgroup data analysis revealed that patients with long-term (> 5 years) diabetes in the non-statin group were more likely to have a higher fibrosis score during the follow-up period compared to statin group patients with a similar DM history. This observation further supports the long-term pleiotropic effects of statins as demonstrated in other diseases, including cancer (Shojaei et al. 2020a). Although, to the best of our knowledge, no publications on the effects of statins on the progression of pulmonary fibrosis in COVID-19 patients are currently available, several clinical and basic science investigations have demonstrated that statins exert significant anti-fibrotic effects in airway resident (mesenchymal) cells and could be beneficial in the treatment of pulmonary disorders characterized by fibrosis (Kou et al. 2022; Schaafsma et al. 2011b). Statins may alleviate post-COVID pulmonary fibrosis by targeting transforming growth factor (TGF)-β signaling, a multifunctional cytokine with profibrogenic effects that is elevated during and after COVID-19 (Pawlos et al. 2021). This cytokine is associated with post-COVID-19 pulmonary fibrosis by promoting lung tissue remodeling and connective tissue deposition among fibroblasts and epithelial cells. On the other hand, statins are believed to suppress epithelial–mesenchymal transition by attenuating TGF-β signaling (Yang et al. 2013). It is also worth mentioning that the effects of statins on fibrosis could, at least in part, be related to the regulation of cellular autophagy (Ghavami et al. 2012, 2014; Shojaei et al.2020a). Indeed, several previous investigations have shown that fibrosis could be regulated via autophagy in various organs, including the lung and heart (Alizadeh et al. 2018; Ghavami et al. 2015, 2018). Recent studies revealed that pulmonary fibrosis is associated with insufficient autophagy, which lead to injury and senescence of alveolar epithelial cells, facilitates epithelial-mesenchymal transformation, and promotes fibroblasts trans-differentiation into myofibroblasts (Araya et al. 2013). Thus, the restoration of impaired autophagy can inhibit fibroblast differentiation and collagen deposition and prevent pulmonary fibrosis, and it has been shown that statins could pulmonary airway inflammation by upregulating autophagy in animal models (Gu et al. 2017). Therefore, the lower pulmonary fibrosis score after the onset of COVID-19 in long-term DM patients on statins may be due to pleiotropic anti-fibrotic effects of statins, possibly through the regulation of autophagy.

The combination of COVID-19 and diabetes could amplify the inflammatory response and contribute to a more severe disease state (Yang et al. 2020). This inflammatory condition is characterized by an increase in serum inflammatory markers, which prognosticate subsequent critical illness in COVID-19 patients. Thus, the empirical findings from our study could be attributed to the well-known anti-inflammatory and immunomodulating effects of statins that are mediated by their impact on immune cells and downregulation of plasma concentrations of inflammatory mediators such as C-reactive protein (CRP), tumor necrosis factor, interleukin (IL)-1, and IL-6 (Ahmadi et al. 2020; Kim et al. 2019; Satny et al. 2021). Baseline laboratory tests indicated that patients in the Non-statin group had significantly higher blood neutrophil and platelet counts. Neutrophils play a crucial role in COVID-19 pathogenesis, particularly in those patients with severe disease courses (Reusch et al. 2021). For example, neutrophils enhance the degranulation of primary granules and promote the release of pro-inflammatory cytokines during SARS-CoV-2 infection (Parackova et al. 2020). Additionally, identified neutrophil activators and effectors were identified as early biomarkers of severe COVID-19 (Meizlish et al. 2021). The inflammatory state is enhanced in DM patients because hyperglycemia induces neutrophils to release neutrophil extracellular traps (NETs), which in turn contribute to the cytokine storm in COVID-19 (Santos et al. 2021). Interestingly, high levels of IL-6 have been shown to induce the systemic release of NETs in other inflammatory diseases of respiratory disease such as severe asthma and chronic obstructive pulmonary disease (Lachowicz-Scroggins et al. 2019; Winslow et al. 2021), and statins may reduce IL-6 release under inflammatory conditions (Loppnow et al. 2011). The possible association between the beneficial effects of statins and IL-6 release in our patients is a subject of our future studies. We also observed a trend for higher median WBC count, hemoglobin, and CRP levels in Non-statin vs Statin patients; however, these apparent differences did not reach statistical significance. In support of our findings, a recent randomized clinical trial revealed that add-on treatment with atorvastatin in hospitalized COVID-19 patients without prior use of statins led to a significant reduction of CRP levels (Davoodi et al. 2021), indicating this might represent one of the possible anti-inflammatory mechanisms of statin therapy in our patient population.

Our research involved a relatively large study population of COVID-19 patients with DM from three hospitals across Iran. Moreover, this is the first prospective study that assessed the effects of statins on pulmonary fibrosis and long-term symptoms of COVID-19. It may provide another useful pleiotropic application of statins and hopefully further future mechanistic investigations open avenues for decreasing the post-COVID-19 effect on the pulmonary functions of DM patients.

## Conclusions

Our work revealed that the use of statins in DM patients with COVID-19 is associated with a lower risk of developing long-term cough and dyspnea. We could not confirm the significant effects of statins on pulmonary fibrosis in our general study population. However, our results do suggest that statins reduce pulmonary fibrosis associated with COVID-19 in long-term (> 5 years) DM patients. Thus, statin therapy appears to be beneficial in DM patients diagnosed with COVID-19, and our findings warrant the pursuit of randomized control trials to verify the therapeutic impact of statin use on clinical outcomes and pulmonary fibrosis in these patients.

## Data Availability

The dataset used to support the findings of this study is available from the corresponding author upon reasoned request.
